# Anaplastic Lymphoma Kinase (ALK)-Rearranged Lung Cancer That Showed Exclusively Scattered Isolated Cells Devoid of Mucin Production in Cytology

**DOI:** 10.7759/cureus.46339

**Published:** 2023-10-01

**Authors:** Tsuyoshi Okazaki, Yoshie Iwasaki, Yuki Kubo, Ken Kodama, Shin-ichi Nakatsuka

**Affiliations:** 1 Department of Clinical Laboratory, Yao Tokushukai General Hospital, Yao, JPN; 2 Department of Pathology, Yao Tokushukai General Hospital, Yao, JPN; 3 Department of Thoracic Surgery, Yao Municipal Hospital, Yao, JPN

**Keywords:** epithelial-mesenchymal transformation, e-cadherin, cytology, lung cancer, alk

## Abstract

We present a rare case of Anaplastic Lymphoma Kinase (*ALK)*-rearranged lung cancer characterized by isolated scattered mucin-free cancer cells forming no clusters in the cytology of endobronchial ultrasound-guided transbronchial needle aspiration (EBUS-TBNA) samples from a paratracheal lymph node. A female patient in her late 40s underwent chest and abdominal CT scan, revealing a 6 cm diameter tumor in the upper lobe of the left lung along with enlargement of mediastinal and hilar lymph nodes, bilateral pleural effusion, and an additional 5.5 cm diameter tumor in the right greater psoas muscle. EBUS-TBNA was performed to obtain samples for cytological and histological examination. Cytology showed exclusively solitary cancer cells that were negative for Periodic Acid-Schiff (PAS) and Alcian blue staining, without clusters. Immunohistochemical analysis of cell block and histology specimens demonstrated positive expression of TTF-1, ALK, and vimentin, while E-cadherin expression was absent. Genetic analysis of samples obtained by EBUS-TBNA confirmed the presence of *EML4-ALK* fusion. The tumor in the right greater psoas muscle was identified as a metastatic tumor from the lung tumor based on ALK-positivity and the *EML4-ALK* fusion. The absence of E-cadherin expression and the presence of vimentin expression suggest that this *ALK*-rearranged lung cancer may have undergone epithelial-mesenchymal transition, resulting in the loss of cellular adhesiveness.

## Introduction

Anaplastic Lymphoma Kinase (*ALK*)-rearranged lung cancer accounts for 2-7% of non-small cell lung cancers [1-3], and is a type of lung cancer for which treatment with ALK tyrosine kinase inhibitors has been proven effective [2,3]. The histological type of *ALK*-rearranged lung cancer is predominantly adenocarcinoma [1,3]. Among the adenocarcinoma subtypes, the solid subtype is more common, while lepidic and acinar subtypes are less frequent as compared to lung adenocarcinomas not associated with *ALK*-rearrangement [4]. Histological characteristics of *ALK*-rearranged lung cancer include cribriform formation and the presence of mucin-producing cells or signet-ring cells as well as psammoma bodies [4-6]. Cytologically *ALK*-rearranged lung cancer is characterized by the frequent presence of signet-ring cells which distribute individually [6-8]. 

Here, we present a rare case of *ALK*-rearranged lung cancer characterized by isolated scattered mucin-free cancer cells forming no clusters in the cytology of endobronchial ultrasound-guided transbronchial needle aspiration (EBUS-TBNA) samples from a paratracheal lymph node.

## Case presentation

Medical history

**Figure 1:**
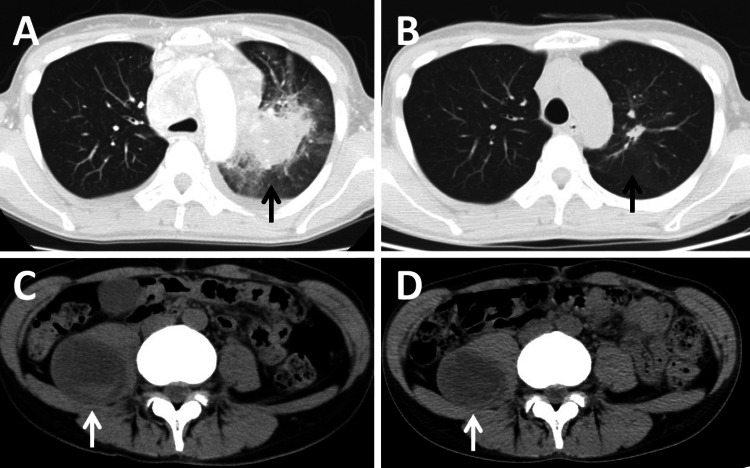
CT scans before and after alectinib treatment A and B, and C and D are CT images of the chest, and the abdomen, respectively. A and C represent CT images prior to the start of alectinib treatment, while B and D are images taken 44 days after treatment initiation. Arrows highlight tumor locations.

A non-smoker female patient in her late 40s presented with cough, sore throat, left anterior chest pain during exhalation, and back pain. Chest and abdominal computed tomography (CT) scans revealed a 6 cm diameter tumor in the upper lobe of the left lung, along with enlargement of mediastinal and hilar lymph nodes, bilateral pleural effusion, and an additional 5.5 cm diameter tumor in the right greater psoas muscle (Figure 1A, 1C). Bronchoscopy indicated narrowing of the trachea due to compression and stenosis of the left and right main bronchi. Endobronchial ultrasound-guided transbronchial needle aspiration (EBUS-TBNA) was performed on the left paratracheal lymph node. Cytological and histological examinations with samples obtained by EBUS-TBNA revealed *ALK*-rearranged lung adenocarcinoma. Cytology of the right pleural fluid also showed ALK-positive cells. Treatment with the ALK tyrosine kinase inhibitor, Alectinib, showed remarkable efficiency and the lung tumor has almost disappeared (Figure 1A, 1B).

However, the tumor in the right greater psoas muscle did not show an apparent reduction, leading to a biopsy guided by CT and subsequent resection of the tumor (Figure 1C, 1D). The tumor was identified as a metastatic tumor from the lung tumor. Despite treatment, cancer recurrence occurred in the lung, and the patient passed away approximately one year after the initial diagnosis.

Cytological findings

**Figure 2:**
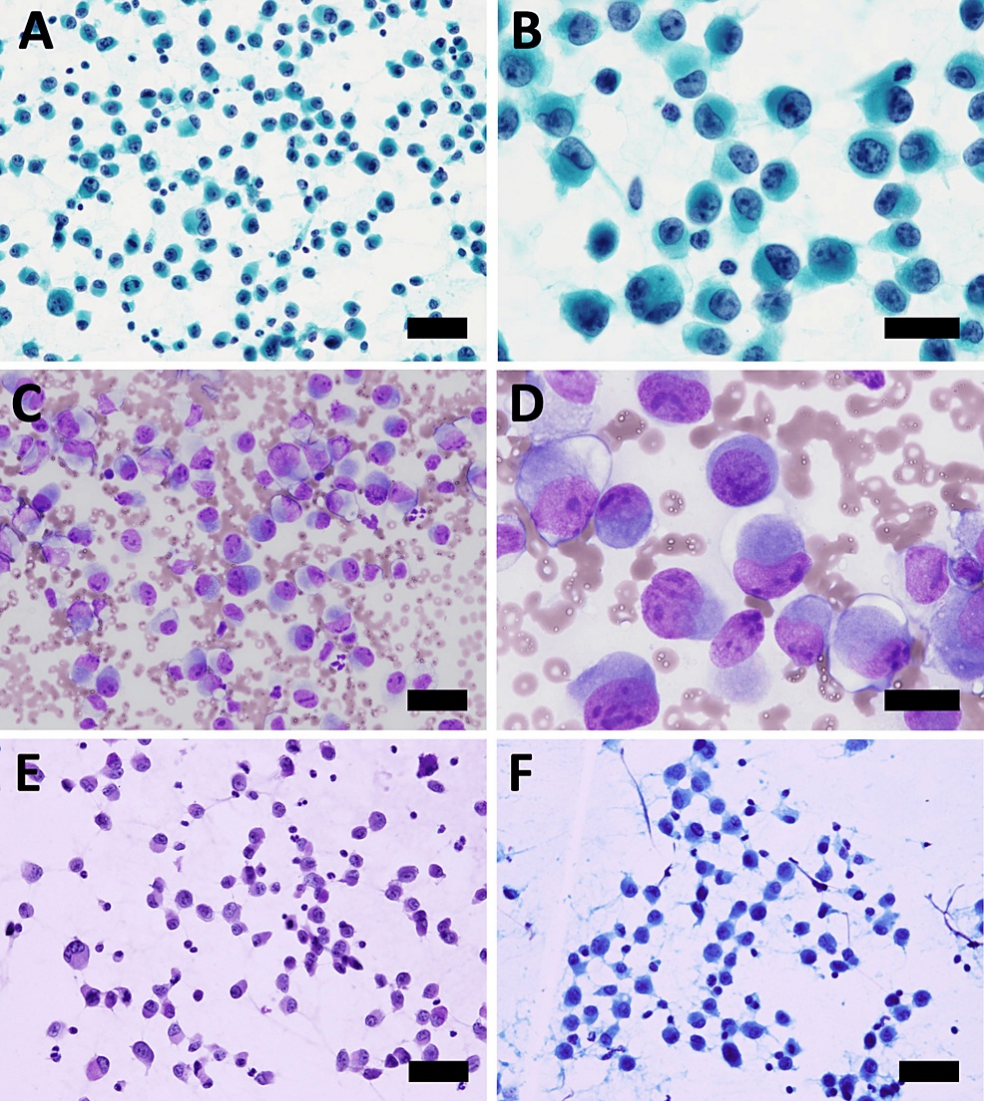
Cytology of samples of a paratracheal lymph node obtained by EBUS-TBNA A and B: Papanicolaou stain, bar 40μm (A) and 20μm (B); C and D: Giemsa stain, x40, bar 40μm (C) and 20μm (D); E: Periodic Acid Schiff (PAS) stain, bar 40μm; F: Alcian blue stain, bar 40μm EBUS-TBNA: endobronchial ultrasound-guided transbronchial needle aspiration

In lymph node EBUS-TBNA cytology, numerous isolated cells were observed without cell clusters (Figure 2).

**Figure 3:**
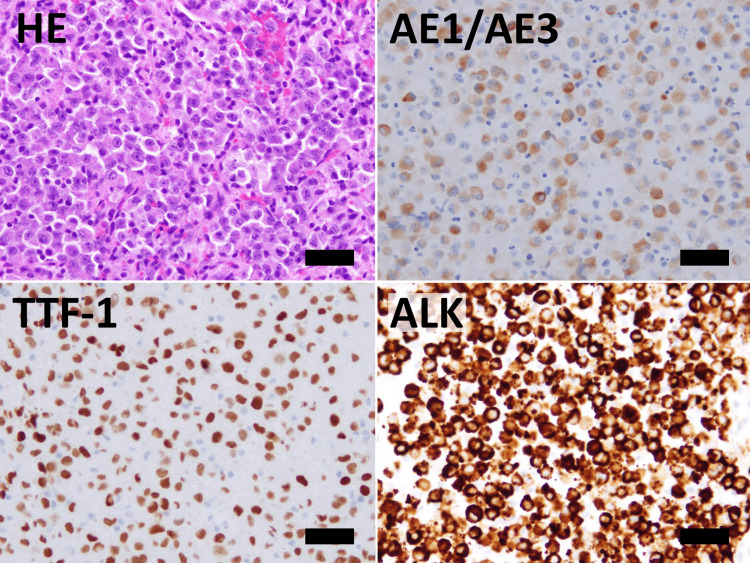
Cell block specimens stained with hematoxylin and eosin (H&E), and immunohistochemically for AE1/AE3 (pankeratin), TTF-1, and ALK. Cancer cells exhibit positive staining for AE1/AE3, TTF-1, and ALK. Bar 40μm.

**Figure 4:**
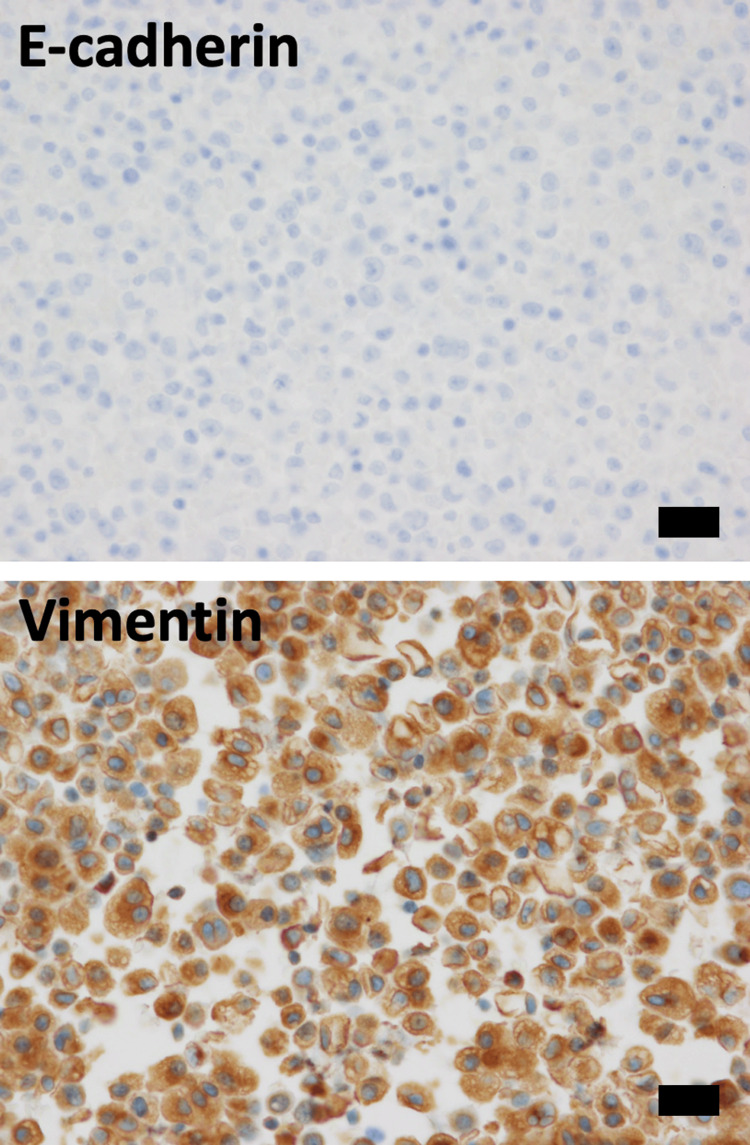
Immunohistochemical staining of cell block specimens for E-cadherin, and vimentin. Cancer cells exhibit negative staining for E-cadherin, and positive staining for vimentin. Bar 50μm.

Cell nuclei were finely granular, exhibiting prominent nucleoli, and an increase in the nuclear-to-cytoplasmic ratio and chromatin. The cytoplasm displayed foamy characteristics with nuclear eccentricity, while periodic acid-Schiff (PAS) and Alcian blue staining were negative. Similar cells were observed in pleural fluid cytology. Cell blocks were prepared using lymph node EBUS-TBNA cytology samples, and immunohistochemical staining was carried out using the BenchMark GX IHC ISH system (F. Hoffmann-La Roche AG, Basel, Switzerland). ALK staining was conducted using the VENTANA OptiView ALK (clone name D5F3) kit (F. Hoffmann-La Roche AG). LCA, CD20, CD79a, CD3, CD56, synaptophysin, chromogranin A, and p40 were all negative, while AE1/AE3, CAM5.2, TTF-1, and ALK were positive (Figure 3). E-cadherin was negative and vimentin was positive (Figure 4).

Histological findings

**Figure 5:**
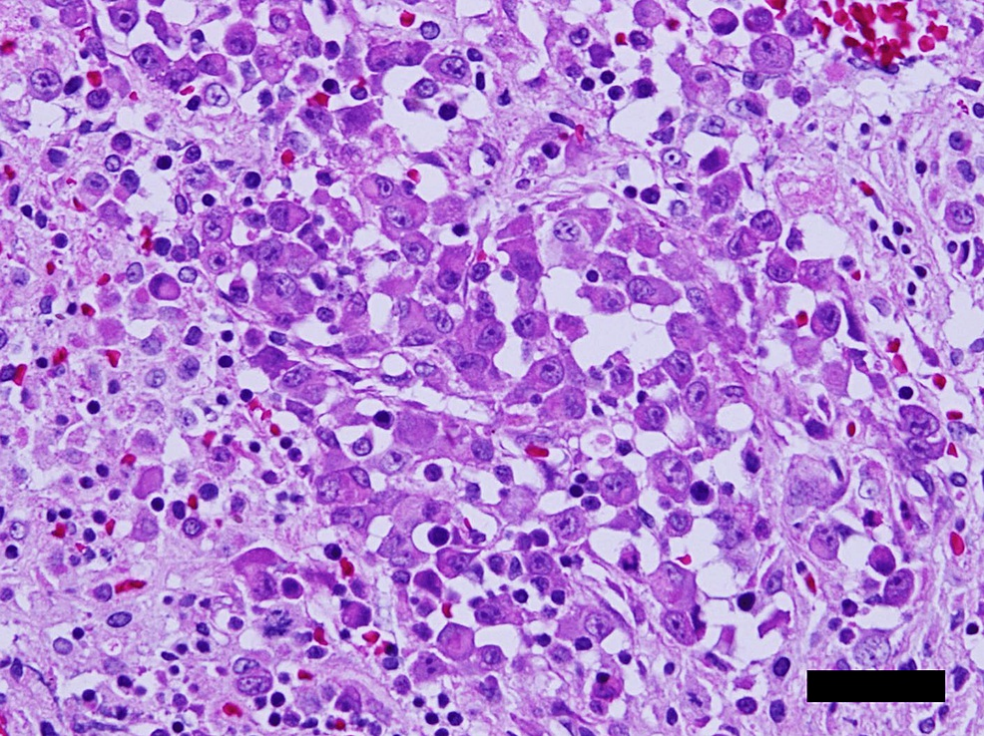
Histology of samples of a paratracheal lymph node obtained by EBUS-TBNA. Hematoxylin and eosin staining. Bar 40μm. EBUS-TBNA: endobronchial ultrasound-guided transbronchial needle aspiration

Immunohistochemistry on cell block specimens from pleural fluid also showed positivity for TTF-1 and ALK. Lymph node EBUS-TBNA tissue specimens showed solid growth of weakly adherent atypical cells exhibiting anisonucleosis with relatively abundant cytoplasm (Figure 5).

Sarcomatous component was not observed. Immunohistochemical results were consistent with those of cell block specimens. Biopsy specimens from the tumor in the right greater psoas muscle showed similar histology (data not shown).

Genetic analysis

**Figure 6:**
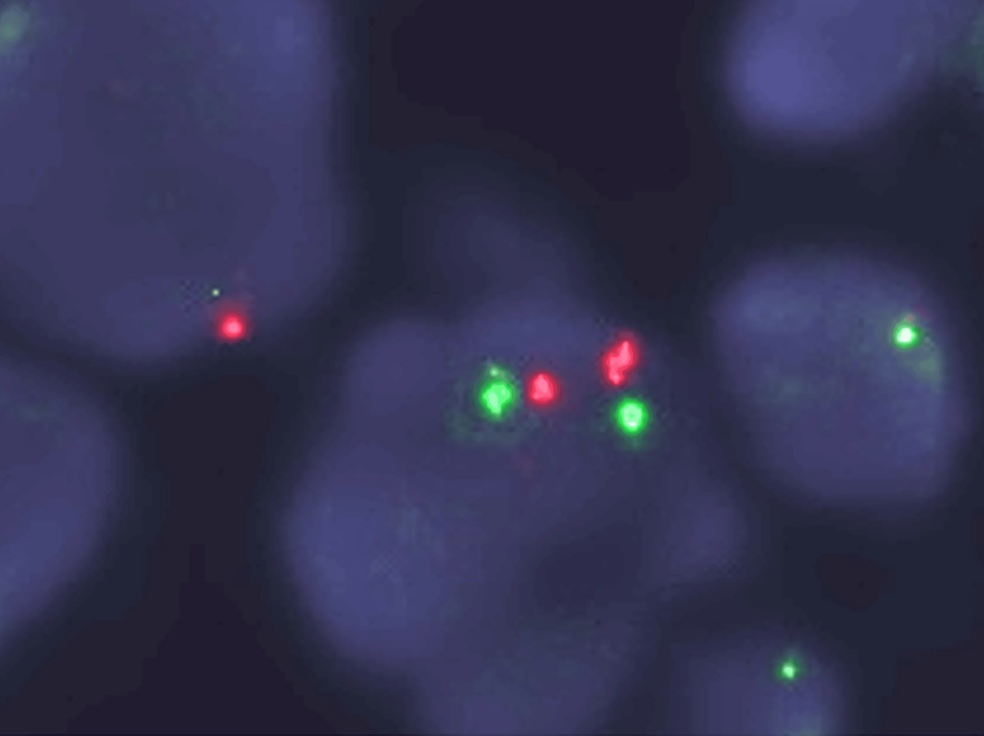
FISH analysis for ALK gene break. FISH analysis was performed using the dual color Vysis ALK Break Apart FISH Probe Kit (Abbott Laboratories, Chicago, Illinois, United States) on lymph node EBUS-TBNA tissue specimens. The presence of red and green spots indicates break of the *ALK* gene. FISH: fluorescence in situ hybridization; EBUS-TBNA: endobronchial ultrasound-guided transbronchial needle aspiration

Fluorescence in situ hybridization (FISH) analysis using the Vysis ALK Break Apart FISH Probe Kit (Abbott Laboratories, Chicago, Illinois, United States) on lymph node EBUS-TBNA tissue specimens demonstrated *ALK*-rearrangement (Figure 6).

**Figure 7:**
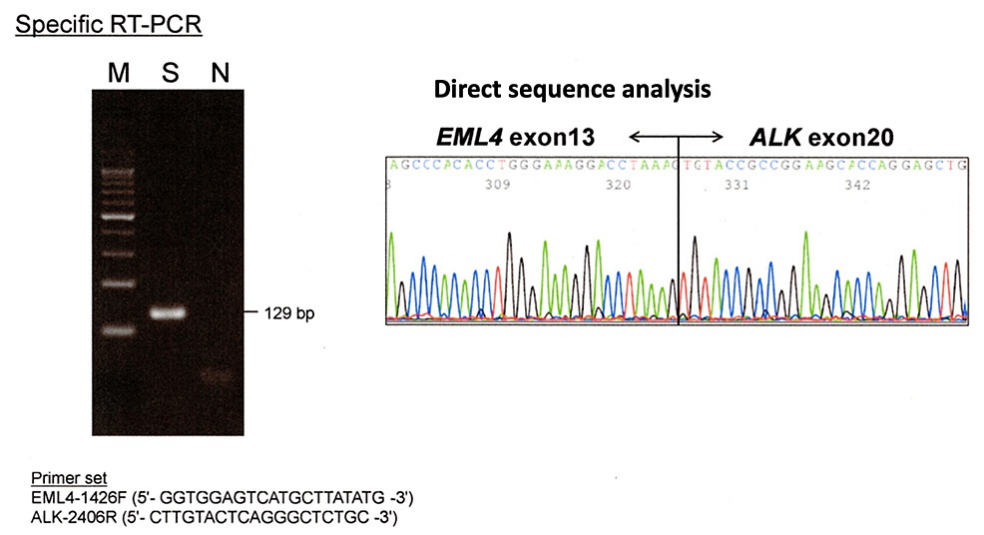
Results of RT-PCR and subsequent direct sequence analysis of PCR amplified fragments using EBUS-TBNA sample. The primer set is the primers for PCR. M; size marker, S; EBUS-TBNA sample, N; sample without *ALK* rearrangement. Direct sequence analysis of 129bp PCR product revealed fusion of *EML4* and *ALK* genes. EBUS-TBNA: endobronchial ultrasound-guided transbronchial needle aspiration; PCR: polymerase chain reaction; RT-PCR: Reverse transcription-polymerase chain reaction

Reverse transcription-polymerase chain reaction (RT-PCR) and subsequent direct sequence analysis of PCR amplified fragments using a lymph node EBUS-TBNA cell block and a tissue from the tumor in the right greater psoas muscle, revealed fusion between exon 13 of *EML4* gene and exon 20 of *ALK* gene (Figure 7).

FISH using the c-met/CEN 7q Dual color FISH probe (GSP Lab Inc., Kobe, Hyogo, Japan) on biopsy tissue specimens from the tumor in the right greater psoas muscle showed about 6.4-fold amplification of the *MET* gene (data not shown). 

## Discussion

The tumor cells in lymph node EBUS-TBNA samples exhibited positivity for epithelial markers (AE1/AE3, and CAM5.2) and TTF-1. Furthermore, the gene analysis revealed *EML4-ALK* fusion. These results indicate that the lung tumor of the present case is a primary *ALK*-rearranged lung adenocarcinoma.

*ALK*-rearranged lung cancers often exhibit the histological subtype of solid adenocarcinoma, frequently showing cribriform pattern, mucin-producing cells, signet-ring cells, and psammoma bodies [4-6] Although the histological type in this case was also solid adenocarcinoma, the histological features reported thus far were not observed. Therefore, this case is not a typical example of *ALK*-rearranged lung cancers reported up to now.

In the cytology of *ALK*-rearranged lung cancer, often there is the presence of signet-ring cells that are distributed solitarily, reflecting its histological characteristics [5-8]. However, in the lymph node EBUS-TBNA cytology of the present case, cancer cells did not exhibit mucin-production cells or signet-ring cells although they were distributed solitarily. Thus, the present case represents a rare type of *ALK*-rearranged lung cancer in that cancer cells produce no mucin, distributed solitarily without exhibiting cell cohesion in its cytology.

In the present case, the absence of cancer cell adhesion in the cytology is likely due to the loss of expression of the cell adhesion factor E-cadherin. Cancer cells were found to express vimentin, a marker of mesenchymal cells. The phenomenon where epithelial cells lose cell adhesion and acquire mesenchymal traits is known as epithelial-mesenchymal transition (EMT) [9,10]. EMT also occurs in cancer cells, allowing them to gain mobility and enhance their invasive abilities [9,10]. In the present case, the cancer cells exhibited markers of EMT, such as the loss of E-cadherin and the positivity of vimentin. Therefore, the loss of cell adhesion suggests the possibility of EMT in the present *ALK*-rearranged lung cancer. Related to this possibility, a previous report has also indicated that *ALK*-rearranged lung cancer has a higher proportion of cases showing decreased E-cadherin expression, or decreased E-cadherin expression along with vimentin positivity, compared to other types of lung cancers, suggesting that *ALK*-rearranged lung cancer is more prone to undergoing epithelial-mesenchymal transition compared to other lung cancer types [4]. Voena et al. showed that epithelial splicing regulatory protein 1, a key regulator of the splicing switch during EMT, was repressed by EML4-ALK activity using *ALK*-rearranged cell lines [11].

When treating *ALK*-rearranged lung cancer with an ALK tyrosine kinase inhibitor, initially, there is a noticeable effect; however, over time, resistance tends to develop [12,13]. Amplification of the *MET* gene accounts for 15% of this resistance [12]. While the primary lung tumor responded to Alectinib (the ALK tyrosine kinase inhibitor) therapy, the metastatic tumor in the right greater psoas muscle exhibited treatment resistance. The resistance appears to stem from *MET* gene amplification, which spontaneously occurred during the metastatic process, as indicated by the metastatic tumor's manifestation of amplified *MET* genes.

## Conclusions

This is a rare case of *ALK*-rearranged lung cancer characterized by isolated scattered mucin-free cancer cells forming no clusters in the cytology of EBUS-TBNA samples from a paratracheal lymph node. The cancer cells exhibited markers of EMT, such as the loss of E-cadherin and the positivity of vimentin. Therefore, the loss of cell adhesion suggests the possibility of EMT in the present *ALK*-rearranged lung cancer.
